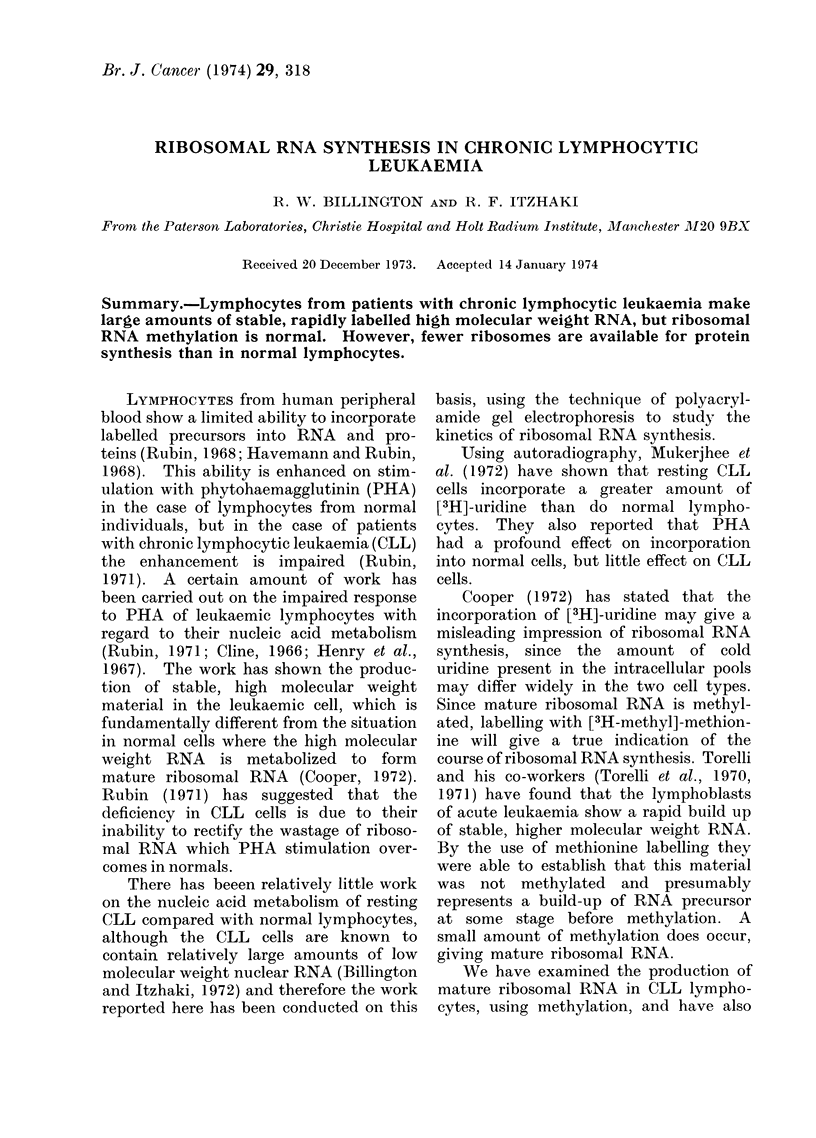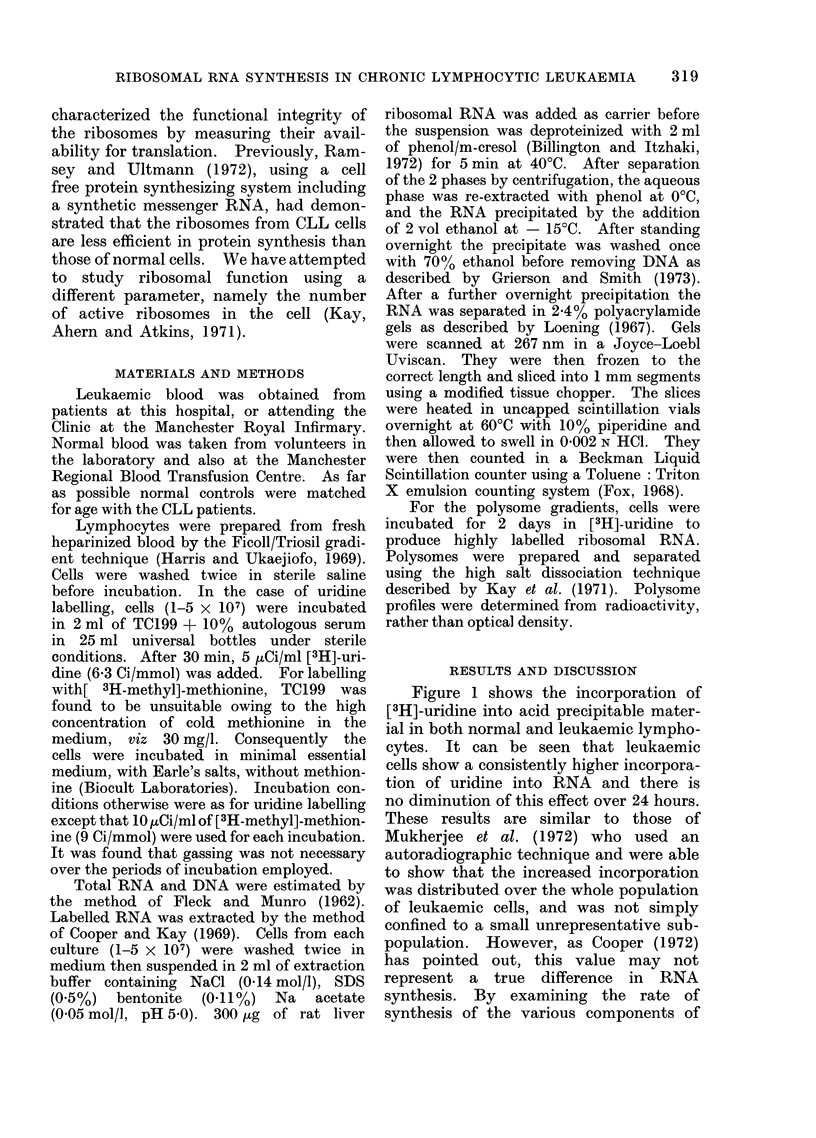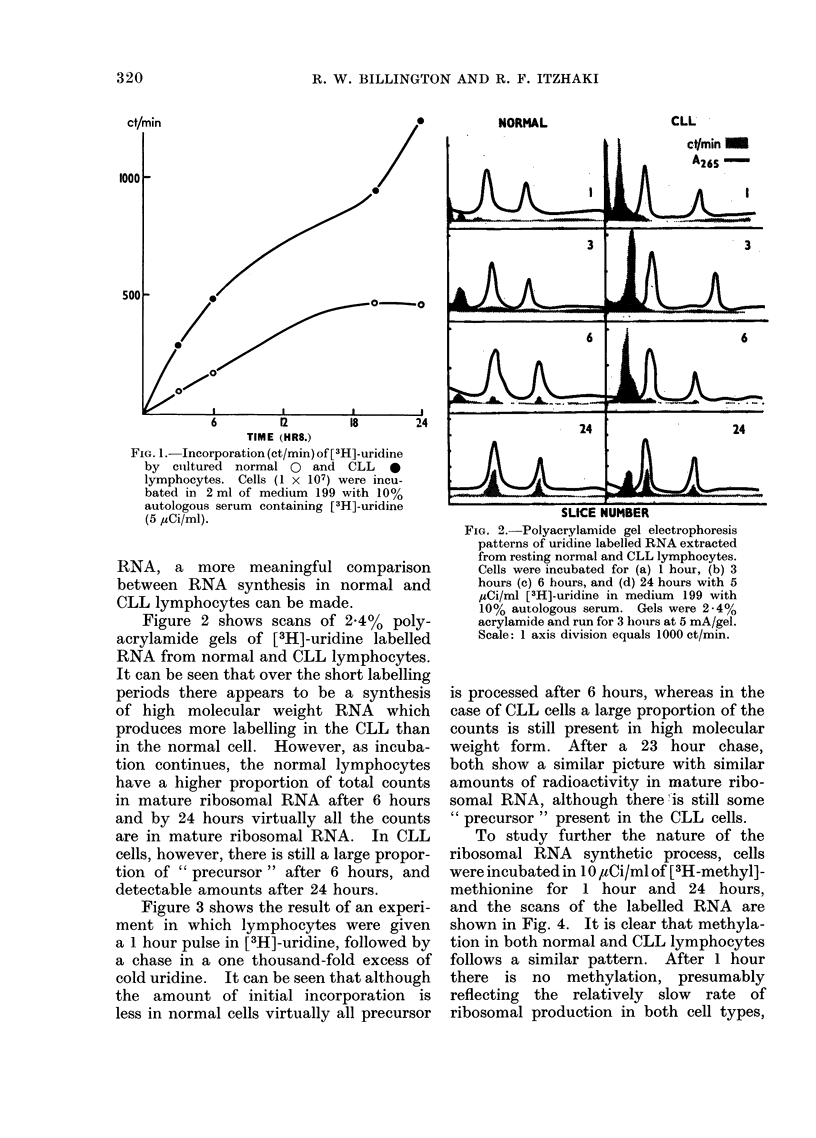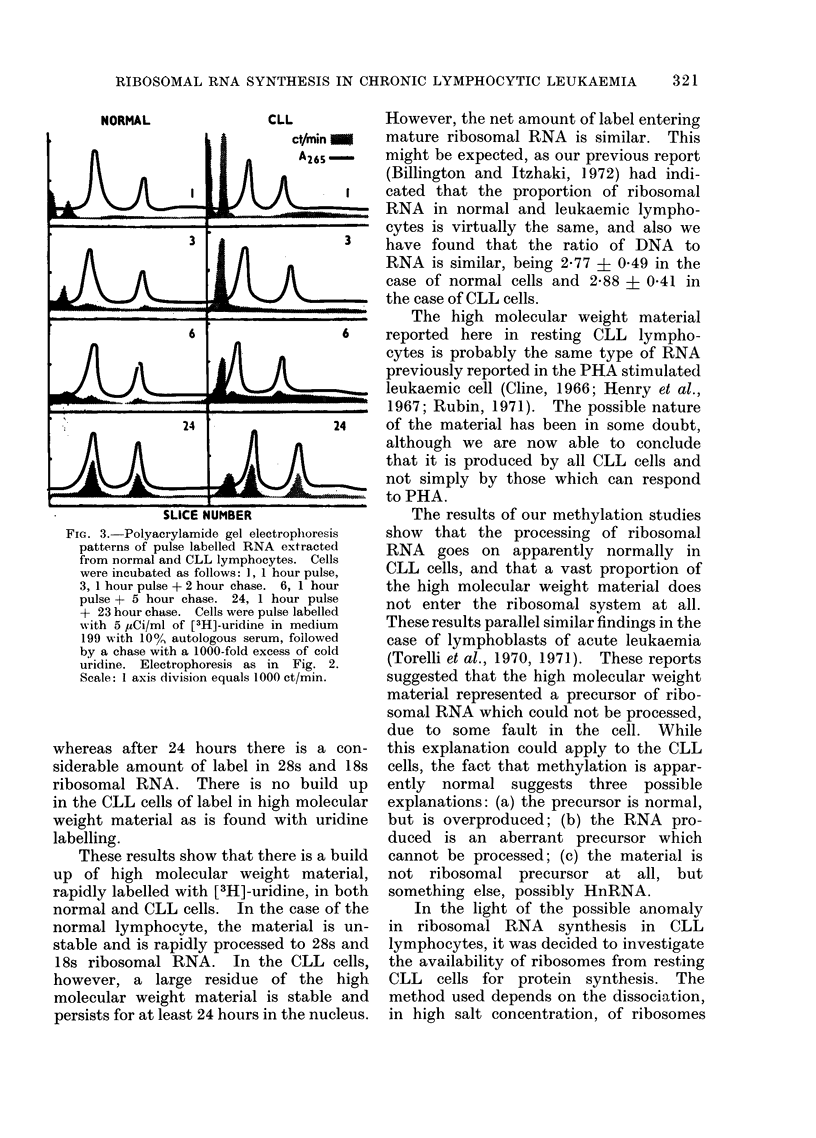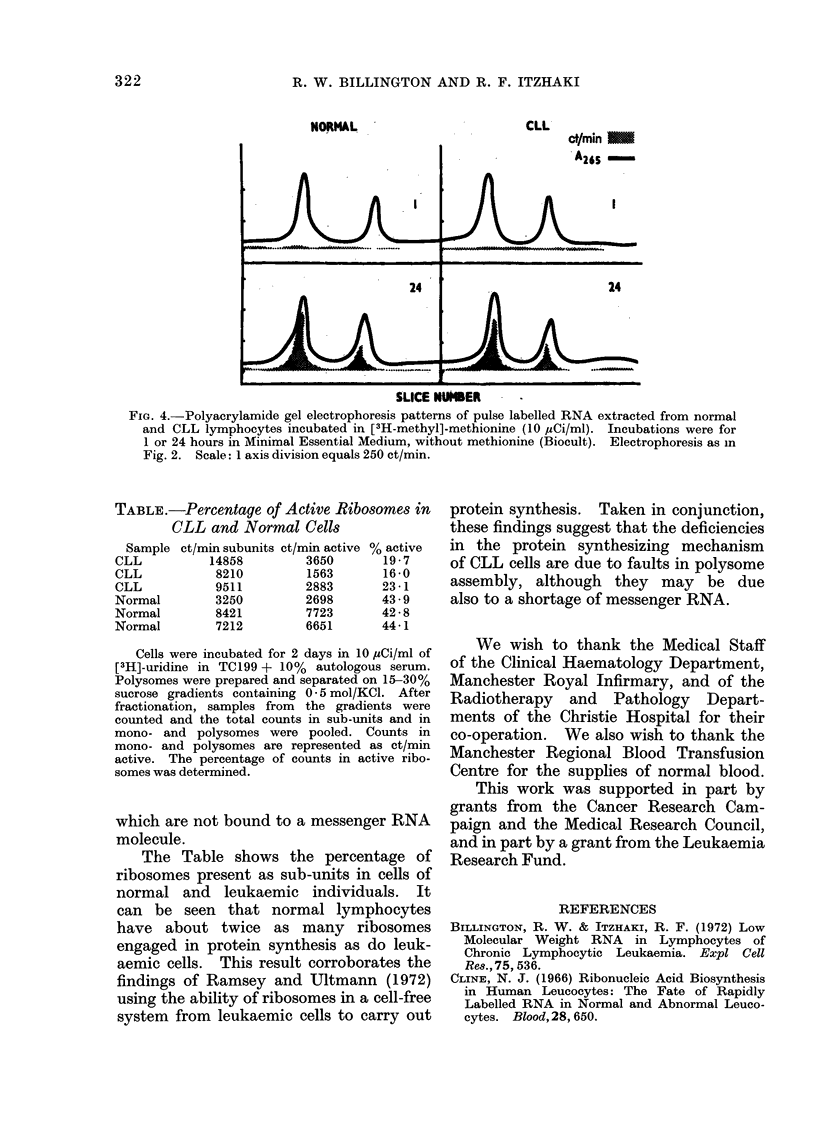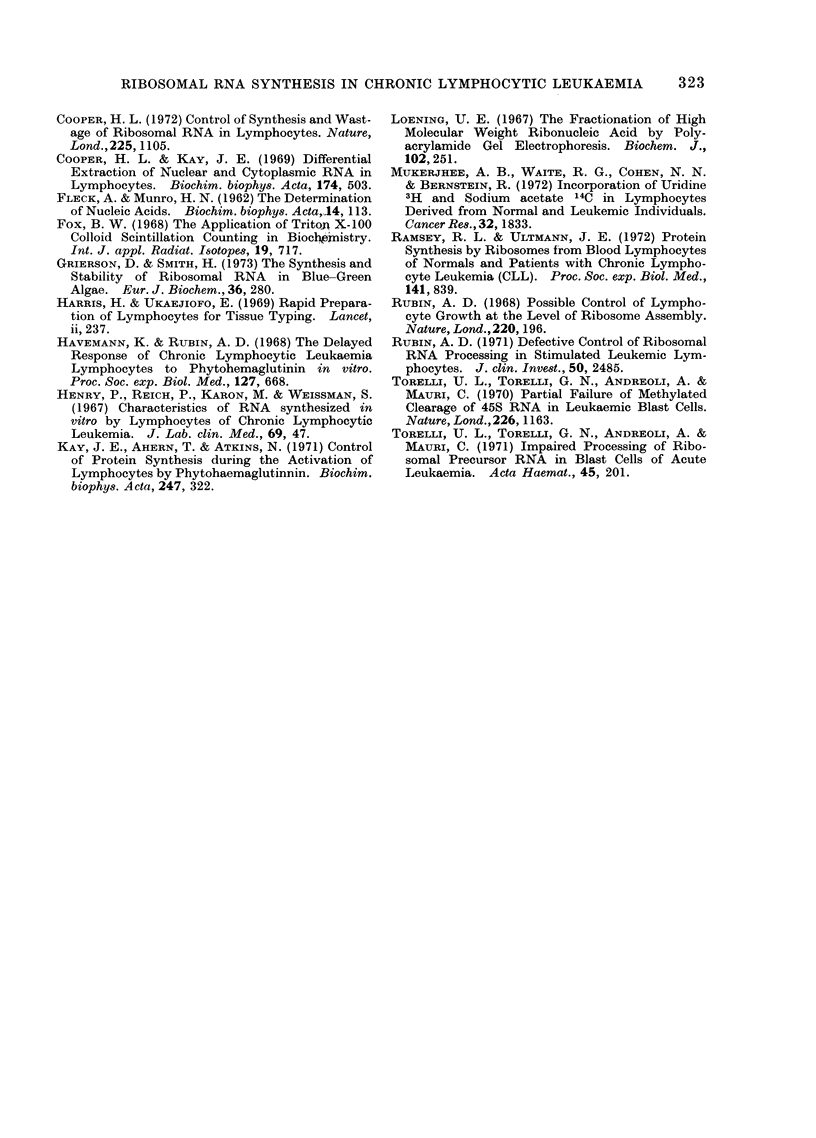# Ribosomal RNA Synthesis in Chronic Lymphocytic Leukaemia

**DOI:** 10.1038/bjc.1974.73

**Published:** 1974-04

**Authors:** R. W. Billington, R. F. Itzhaki

## Abstract

Lymphocytes from patients with chronic lymphocytic leukaemia make large amounts of stable, rapidly labelled high molecular weight RNA, but ribosomal RNA methylation is normal. However, fewer ribosomes are available for protein synthesis than in normal lymphocytes.


*Br. J. Cancer* (1974) **29**, 318

### RIBOSOMAL RNA SYNTHESIS IN CHRONIC LYMPHOCYTIC LEUKAEMIA

R. W. BILLINGTON AND R. F. ITZHAKI

*From the Paterson Laboratories, Christie Hospital and Holt Radium Institute, Manchester M20 9BX*

Received 20 December 1973. Accepted 14 January 1974

**Summary.—Lymphocytes from patients with chronic lymphocytic leukaemia make large amounts of stable, rapidly labelled high molecular weight RNA, but ribosomal RNA methylation is normal. However, fewer ribosomes are available for protein synthesis than in normal lymphocytes.**

LYMPHOCYTES from human peripheral blood show a limited ability to incorporate labelled precursors into RNA and proteins (Rubin, 1968; Havemann and Rubin, 1968). This ability is enhanced on stimulation with phytohaemagglutinin (PHA) in the case of lymphocytes from normal individuals, but in the case of patients with chronic lymphocytic leukaemia (CLL) the enhancement is impaired (Rubin, 1971). A certain amount of work has been carried out on the impaired response to PHA of leukaemic lymphocytes with regard to their nucleic acid metabolism (Rubin, 1971; Cline, 1966; Henry *et al.*, 1967). The work has shown the production of stable, high molecular weight material in the leukaemic cell, which is fundamentally different from the situation in normal cells where the high molecular weight RNA is metabolized to form mature ribosomal RNA (Cooper, 1972). Rubin (1971) has suggested that the deficiency in CLL cells is due to their inability to rectify the wastage of ribosomal RNA which PHA stimulation overcomes in normals.

There has beeen relatively little work on the nucleic acid metabolism of resting CLL compared with normal lymphocytes, although the CLL cells are known to contain relatively large amounts of low molecular weight nuclear RNA (Billington and Itzhaki, 1972) and therefore the work reported here has been conducted on this basis, using the technique of polyacrylamide gel electrophoresis to study the kinetics of ribosomal RNA synthesis.

Using autoradiography, Mukerjhee *et al.* (1972) have shown that resting CLL cells incorporate a greater amount of [$^3$H]-uridine than do normal lymphocytes. They also reported that PHA had a profound effect on incorporation into normal cells, but little effect on CLL cells.

Cooper (1972) has stated that the incorporation of [$^3$H]-uridine may give a misleading impression of ribosomal RNA synthesis, since the amount of cold uridine present in the intracellular pools may differ widely in the two cell types. Since mature ribosomal RNA is methylated, labelling with [$^3$H-methyl]-methionine will give a true indication of the course of ribosomal RNA synthesis. Torelli and his co-workers (Torelli *et al.*, 1970, 1971) have found that the lymphoblasts of acute leukaemia show a rapid build up of stable, higher molecular weight RNA. By the use of methionine labelling they were able to establish that this material was not methylated and presumably represents a build-up of RNA precursor at some stage before methylation. A small amount of methylation does occur, giving mature ribosomal RNA.

We have examined the production of mature ribosomal RNA in CLL lymphocytes, using methylation, and have also characterized the functional integrity of the ribosomes by measuring their availability for translation. Previously, Ramsey and Ultmann (1972), using a cell free protein synthesizing system including a synthetic messenger RNA, had demonstrated that the ribosomes from CLL cells are less efficient in protein synthesis than those of normal cells. We have attempted to study ribosomal function using a different parameter, namely the number of active ribosomes in the cell (Kay, Ahern and Atkins, 1971).

### MATERIALS AND METHODS

Leukaemic blood was obtained from patients at this hospital, or attending the Clinic at the Manchester Royal Infirmary. Normal blood was taken from volunteers in the laboratory and also at the Manchester Regional Blood Transfusion Centre. As far as possible normal controls were matched for age with the CLL patients.

Lymphocytes were prepared from fresh heparinized blood by the Ficoll/Triosil gradient technique (Harris and Ukaejiofo, 1969). Cells were washed twice in sterile saline before incubation. In the case of uridine labelling, cells ($1–5 \times 10^7$) were incubated in 2 ml of TC199 + 10% autologous serum in 25 ml universal bottles under sterile conditions. After 30 min, 5 $\mu$Ci/ml [$^3$H]-uridine (6·3 Ci/mmol) was added. For labelling with[ $^3$H-methyl]-methionine, TC199 was found to be unsuitable owing to the high concentration of cold methionine in the medium, *viz* 30 mg/l. Consequently the cells were incubated in minimal essential medium, with Earle's salts, without methionine (Biocult Laboratories). Incubation conditions otherwise were as for uridine labelling except that 10 $\mu$Ci/ml of [$^3$H-methyl]-methionine (9 Ci/mmol) were used for each incubation. It was found that gassing was not necessary over the periods of incubation employed.

Total RNA and DNA were estimated by the method of Fleck and Munro (1962). Labelled RNA was extracted by the method of Cooper and Kay (1969). Cells from each culture ($1–5 \times 10^7$) were washed twice in medium then suspended in 2 ml of extraction buffer containing NaCl (0·14 mol/l), SDS (0·5%) bentonite (0·11%) Na acetate (0·05 mol/l, pH 5·0). 300 $\mu$g of rat liver ribosomal RNA was added as carrier before the suspension was deproteinized with 2 ml of phenol/m-cresol (Billington and Itzhaki, 1972) for 5 min at 40°C. After separation of the 2 phases by centrifugation, the aqueous phase was re-extracted with phenol at 0°C, and the RNA precipitated by the addition of 2 vol ethanol at − 15°C. After standing overnight the precipitate was washed once with 70% ethanol before removing DNA as described by Grierson and Smith (1973). After a further overnight precipitation the RNA was separated in 2·4% polyacrylamide gels as described by Loening (1967). Gels were scanned at 267 nm in a Joyce–Loebl Uviscan. They were then frozen to the correct length and sliced into 1 mm segments using a modified tissue chopper. The slices were heated in uncapped scintillation vials overnight at 60°C with 10% piperidine and then allowed to swell in 0·002 N HCl. They were then counted in a Beckman Liquid Scintillation counter using a Toluene : Triton X emulsion counting system (Fox, 1968).

For the polysome gradients, cells were incubated for 2 days in [$^3$H]-uridine to produce highly labelled ribosomal RNA. Polysomes were prepared and separated using the high salt dissociation technique described by Kay *et al.* (1971). Polysome profiles were determined from radioactivity, rather than optical density.

### RESULTS AND DISCUSSION

Figure 1 shows the incorporation of [$^3$H]-uridine into acid precipitable material in both normal and leukaemic lymphocytes. It can be seen that leukaemic cells show a consistently higher incorporation of uridine into RNA and there is no diminution of this effect over 24 hours. These results are similar to those of Mukherjee *et al.* (1972) who used an autoradiographic technique and were able to show that the increased incorporation was distributed over the whole population of leukaemic cells, and was not simply confined to a small unrepresentative subpopulation. However, as Cooper (1972) has pointed out, this value may not represent a true difference in RNA synthesis. By examining the rate of synthesis of the various components of

FIG. 1.—Incorporation (ct/min) of [$^3$H]-uridine by cultured normal ○ and CLL ● lymphocytes. Cells (1 × $10^7$) were incubated in 2 ml of medium 199 with 10% autologous serum containing [$^3$H]-uridine (5 $\mu$Ci/ml).

FIG. 2.—Polyacrylamide gel electrophoresis patterns of uridine labelled RNA extracted from resting normal and CLL lymphocytes. Cells were incubated for (a) 1 hour, (b) 3 hours (c) 6 hours, and (d) 24 hours with 5 $\mu$Ci/ml [$^3$H]-uridine in medium 199 with 10% autologous serum. Gels were 2·4% acrylamide and run for 3 hours at 5 mA/gel. Scale: 1 axis division equals 1000 ct/min.

RNA, a more meaningful comparison between RNA synthesis in normal and CLL lymphocytes can be made.

Figure 2 shows scans of 2·4% polyacrylamide gels of [$^3$H]-uridine labelled RNA from normal and CLL lymphocytes. It can be seen that over the short labelling periods there appears to be a synthesis of high molecular weight RNA which produces more labelling in the CLL than in the normal cell. However, as incubation continues, the normal lymphocytes have a higher proportion of total counts in mature ribosomal RNA after 6 hours and by 24 hours virtually all the counts are in mature ribosomal RNA. In CLL cells, however, there is still a large proportion of " precursor " after 6 hours, and detectable amounts after 24 hours.

Figure 3 shows the result of an experiment in which lymphocytes were given a 1 hour pulse in [$^3$H]-uridine, followed by a chase in a one thousand-fold excess of cold uridine. It can be seen that although the amount of initial incorporation is less in normal cells virtually all precursor is processed after 6 hours, whereas in the case of CLL cells a large proportion of the counts is still present in high molecular weight form. After a 23 hour chase, both show a similar picture with similar amounts of radioactivity in mature ribosomal RNA, although there is still some " precursor " present in the CLL cells.

To study further the nature of the ribosomal RNA synthetic process, cells were incubated in 10 $\mu$Ci/ml of [$^3$H-methyl]-methionine for 1 hour and 24 hours, and the scans of the labelled RNA are shown in Fig. 4. It is clear that methylation in both normal and CLL lymphocytes follows a similar pattern. After 1 hour there is no methylation, presumably reflecting the relatively slow rate of ribosomal production in both cell types,

FIG. 3.—Polyacrylamide gel electrophoresis patterns of pulse labelled RNA extracted from normal and CLL lymphocytes. Cells were incubated as follows: 1, 1 hour pulse, 3, 1 hour pulse + 2 hour chase. 6, 1 hour pulse + 5 hour chase. 24, 1 hour pulse + 23 hour chase. Cells were pulse labelled with 5 $\mu$Ci/ml of [$^3$H]-uridine in medium 199 with 10% autologous serum, followed by a chase with a 1000-fold excess of cold uridine. Electrophoresis as in Fig. 2. Scale: 1 axis division equals 1000 ct/min.

whereas after 24 hours there is a considerable amount of label in 28s and 18s ribosomal RNA. There is no build up in the CLL cells of label in high molecular weight material as is found with uridine labelling.

These results show that there is a build up of high molecular weight material, rapidly labelled with [$^3$H]-uridine, in both normal and CLL cells. In the case of the normal lymphocyte, the material is unstable and is rapidly processed to 28s and 18s ribosomal RNA. In the CLL cells, however, a large residue of the high molecular weight material is stable and persists for at least 24 hours in the nucleus. However, the net amount of label entering mature ribosomal RNA is similar. This might be expected, as our previous report (Billington and Itzhaki, 1972) had indicated that the proportion of ribosomal RNA in normal and leukaemic lymphocytes is virtually the same, and also we have found that the ratio of DNA to RNA is similar, being 2·77 ± 0·49 in the case of normal cells and 2·88 ± 0·41 in the case of CLL cells.

The high molecular weight material reported here in resting CLL lymphocytes is probably the same type of RNA previously reported in the PHA stimulated leukaemic cell (Cline, 1966; Henry *et al.*, 1967; Rubin, 1971). The possible nature of the material has been in some doubt, although we are now able to conclude that it is produced by all CLL cells and not simply by those which can respond to PHA.

The results of our methylation studies show that the processing of ribosomal RNA goes on apparently normally in CLL cells, and that a vast proportion of the high molecular weight material does not enter the ribosomal system at all. These results parallel similar findings in the case of lymphoblasts of acute leukaemia (Torelli *et al.*, 1970, 1971). These reports suggested that the high molecular weight material represented a precursor of ribosomal RNA which could not be processed, due to some fault in the cell. While this explanation could apply to the CLL cells, the fact that methylation is apparently normal suggests three possible explanations: (a) the precursor is normal, but is overproduced; (b) the RNA produced is an aberrant precursor which cannot be processed; (c) the material is not ribosomal precursor at all, but something else, possibly HnRNA.

In the light of the possible anomaly in ribosomal RNA synthesis in CLL lymphocytes, it was decided to investigate the availability of ribosomes from resting CLL cells for protein synthesis. The method used depends on the dissociation, in high salt concentration, of ribosomes

FIG. 4.—Polyacrylamide gel electrophoresis patterns of pulse labelled RNA extracted from normal and CLL lymphocytes incubated in [$^3$H-methyl]-methionine (10 $\mu$Ci/ml). Incubations were for 1 or 24 hours in Minimal Essential Medium, without methionine (Biocult). Electrophoresis as in Fig. 2. Scale: 1 axis division equals 250 ct/min.

TABLE.—*Percentage of Active Ribosomes in CLL and Normal Cells*

| Sample | ct/min subunits | ct/min active | % active |
|---|---|---|---|
| CLL | 14858 | 3650 | 19·7 |
| CLL | 8210 | 1563 | 16·0 |
| CLL | 9511 | 2883 | 23·1 |
| Normal | 3250 | 2698 | 43·9 |
| Normal | 8421 | 7723 | 42·8 |
| Normal | 7212 | 6651 | 44·1 |

Cells were incubated for 2 days in 10 $\mu$Ci/ml of [$^3$H]-uridine in TC199 + 10% autologous serum. Polysomes were prepared and separated on 15–30% sucrose gradients containing 0·5 mol/KCl. After fractionation, samples from the gradients were counted and the total counts in sub-units and in mono- and polysomes were pooled. Counts in mono- and polysomes are represented as ct/min active. The percentage of counts in active ribosomes was determined.

which are not bound to a messenger RNA molecule.

The Table shows the percentage of ribosomes present as sub-units in cells of normal and leukaemic individuals. It can be seen that normal lymphocytes have about twice as many ribosomes engaged in protein synthesis as do leukaemic cells. This result corroborates the findings of Ramsey and Ultmann (1972) using the ability of ribosomes in a cell-free system from leukaemic cells to carry out protein synthesis. Taken in conjunction, these findings suggest that the deficiencies in the protein synthesizing mechanism of CLL cells are due to faults in polysome assembly, although they may be due also to a shortage of messenger RNA.

We wish to thank the Medical Staff of the Clinical Haematology Department, Manchester Royal Infirmary, and of the Radiotherapy and Pathology Departments of the Christie Hospital for their co-operation. We also wish to thank the Manchester Regional Blood Transfusion Centre for the supplies of normal blood.

This work was supported in part by grants from the Cancer Research Campaign and the Medical Research Council, and in part by a grant from the Leukaemia Research Fund.

REFERENCES

BILLINGTON, R. W. & ITZHAKI, R. F. (1972) Low Molecular Weight RNA in Lymphocytes of Chronic Lymphocytic Leukaemia. *Expl Cell Res.*, **75**, 536.

CLINE, N. J. (1966) Ribonucleic Acid Biosynthesis in Human Leucocytes: The Fate of Rapidly Labelled RNA in Normal and Abnormal Leucocytes. *Blood*, **28**, 650.

COOPER, H. L. (1972) Control of Synthesis and Wastage of Ribosomal RNA in Lymphocytes. *Nature, Lond.*, **225**, 1105.

COOPER, H. L. & KAY, J. E. (1969) Differential Extraction of Nuclear and Cytoplasmic RNA in Lymphocytes. *Biochim. biophys. Acta*, **174**, 503.

FLECK, A. & Munro, H. N. (1962) The Determination of Nucleic Acids. *Biochim. biophys. Acta*, **14**, 113.

FOX, B. W. (1968) The Application of Triton X-100 Colloid Scintillation Counting in Biochemistry. *Int. J. appl. Radiat. Isotopes*, **19**, 717.

GRIERSON, D. & SMITH, H. (1973) The Synthesis and Stability of Ribosomal RNA in Blue–Green Algae. *Eur. J. Biochem.*, **36**, 280.

HARRIS, H. & UKAEJIOFO, E. (1969) Rapid Preparation of Lymphocytes for Tissue Typing. *Lancet*, ii, 237.

HAVEMANN, K. & RUBIN, A. D. (1968) The Delayed Response of Chronic Lymphocytic Leukaemia Lymphocytes to Phytohemaglutinin *in vitro*. *Proc. Soc. exp. Biol. Med.*, **127**, 668.

HENRY, P., REICH, P., KARON, M. & WEISSMAN, S. (1967) Characteristics of RNA synthesized *in vitro* by Lymphocytes of Chronic Lymphocytic Leukemia. *J. Lab. clin. Med.*, **69**, 47.

KAY, J. E., AHERN, T. & ATKINS, N. (1971) Control of Protein Synthesis during the Activation of Lymphocytes by Phytohaemaglutinnin. *Biochim. biophys. Acta*, **247**, 322.

LOENING, U. E. (1967) The Fractionation of High Molecular Weight Ribonucleic Acid by Polyacrylamide Gel Electrophoresis. *Biochem. J.*, **102**, 251.

MUKERJHEE, A. B., WAITE, R. G., COHEN, N. N. & BERNSTEIN, R. (1972) Incorporation of Uridine $^{3}H$ and Sodium acetate $^{14}C$ in Lymphocytes Derived from Normal and Leukemic Individuals. *Cancer Res.*, **32**, 1833.

RAMSEY, R. L. & ULTMANN, J. E. (1972) Protein Synthesis by Ribosomes from Blood Lymphocytes of Normals and Patients with Chronic Lymphocyte Leukemia (CLL). *Proc. Soc. exp. Biol. Med.*, **141**, 839.

RUBIN, A. D. (1968) Possible Control of Lymphocyte Growth at the Level of Ribosome Assembly. *Nature, Lond.*, **220**, 196.

RUBIN, A. D. (1971) Defective Control of Ribosomal RNA Processing in Stimulated Leukemic Lymphocytes. *J. clin. Invest.*, **50**, 2485.

TORELLI, U. L., TORELLI, G. N., ANDREOLI, A. & MAURI, C. (1970) Partial Failure of Methylated Clearage of 45S RNA in Leukaemic Blast Cells. *Nature, Lond.*, **226**, 1163.

TORELLI, U. L., TORELLI, G. N., ANDREOLI, A. & MAURI, C. (1971) Impaired Processing of Ribosomal Precursor RNA in Blast Cells of Acute Leukaemia. *Acta Haemat.*, **45**, 201.